# Yeast 2.0—connecting the dots in the construction of the world's first functional synthetic eukaryotic genome

**DOI:** 10.1093/femsyr/foy032

**Published:** 2018-03-16

**Authors:** I S Pretorius, J D Boeke

**Affiliations:** 1 Chancellery, Macquarie University, Sydney, NSW 2109, Australia; 2 Institute for Systems Genetics and Department of Biochemistry and Molecular Pharmacology, NYU Langone Health, New York, NY 10016, USA

**Keywords:** biodesign, bioengineering, genome engineering, *Saccharomyces cerevisiae*, Sc2.0, synthetic biology, synthetic chromosomes, synthetic genome, yeast 2.0

## Abstract

Historians of the future may well describe 2018 as the year that the world's first functional synthetic eukaryotic genome became a reality. Without the benefit of hindsight, it might be hard to completely grasp the long-term significance of a breakthrough moment in the history of science like this. The role of synthetic biology in the imminent birth of a budding *Saccharomyces cerevisiae* yeast cell carrying 16 man-made chromosomes causes the world of science to teeter on the threshold of a future-defining scientific frontier. The genome-engineering tools and technologies currently being developed to produce the ultimate yeast genome will irreversibly connect the dots between our improved understanding of the fundamentals of a complex cell containing its DNA in a specialised nucleus and the application of bioengineered eukaryotes designed for advanced biomanufacturing of beneficial products. By joining up the dots between the findings and learnings from the international *Synthetic Yeast Genome* project (known as the *Yeast 2.0* or *Sc2.0* project) and concurrent advancements in biodesign tools and smart data-intensive technologies, a future world powered by a thriving bioeconomy seems realistic. This global project demonstrates how a collaborative network of dot connectors—driven by a tinkerer's indomitable curiosity to understand how things work inside a eukaryotic cell—are using cutting-edge biodesign concepts and synthetic biology tools to advance science and to positively frame human futures (i.e. improved quality of life) in a planetary context (i.e. a sustainable environment). Explorations such as this have a rich history of resulting in unexpected discoveries and unanticipated applications for the benefit of people and planet. However, we must learn from past explorations into controversial futuristic sciences and ensure that researchers at the forefront of an emerging science such as synthetic biology remain connected to all stakeholders’ concerns about the biosafety, bioethics and regulatory aspects of their pioneering work. This article presents a shared vision of constructing a synthetic eukaryotic genome in a safe model organism by using novel concepts and advanced technologies. This multidisciplinary and collaborative project is conducted under a sound governance structure that does not only respect the scientific achievements and lessons from the past, but that is also focussed on leading the present and helping to secure a brighter future for all.

## Teaser

The international Synthetic Yeast Genome (Sc2.0) project is discovering the dots that connect basic eukaryotic biology, biodesign, bioengineering and biomanufacturing with a budding bioeconomy of the future.

## THE CONNECTION BETWEEN SYNTHETIC BIOLOGY AND YEAST

### Synthetic biology—a dot-connecting science with boundless potential

**Figure 1.**
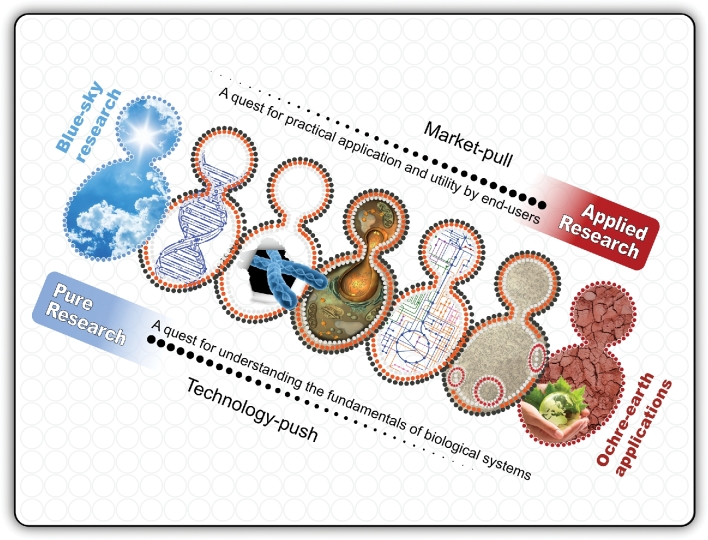
The connection between ‘blue-sky’ yeast research and ‘ochre-earth’ practical applications. At the ‘blue-sky’ end of the spectrum, *pure* or *basic* research is inspired by curiosity and a quest for understanding the fundamental intricacies of a yeast cell's inner workings. Explorations into the fundamentals of cells and organisms are often supported and pushed by technological advances. At the ‘ochre-earth’ side of the spectrum, *applied* or *goal-oriented* research is usually pulled by market forces and the requirements of end users. Research inspired by both the quest for understanding the ‘fundamentals’ and the promise of future ‘use’ often provides the most powerful dynamo of technological and societal progress. This approach is particularly relevant to yeast research as *Saccharomyces cerevisiae* is both an ideal model organism in the laboratory and a workhorse for several fermentation-based industries.

In the era of modern science, hardly a week goes by without a breakthrough discovery somewhere in the world in what is colloquially termed as ‘blue-sky’ research. Blue-sky thinking and imagination are scientists’ way of shedding light on the dim places where reason itself often has yet to voyage. However, there is often a disconnect between scientists’ excitement about the basic ‘blue-sky’ discoveries in their research laboratories and the ‘down-to-ochre-earth’ perceptions of those discoveries in ordinary households. This disconnect between ‘blue-sky’ science and ‘ochre-earth’ interpretations by the average person frequently throws science into a maelstrom of media frenzies, political point-scoring debates and ‘hype-horror-hope’ conversations around boardroom tables and barbeque fires. Confused and, at times, frustrated, many tax-paying citizens and sceptical voters query the connection between *basic research* (i.e. ‘pure’ research inspired by curiosity and a quest for fundamental understanding) and goal-oriented *applied research* (i.e. ‘practical’ research inspired by utility, and from the outset, focussed on beneficial outcomes for end users in industry and society at large). Simply put, scientists—anchored in the ‘ochre-earth’ needs of society and reaching across the horizon for the ‘blue-skies’ of new discoveries and innovation—bear the responsibility to continuously brighten up the ‘dull-beige’ patch of ignorance and confusion that can lie between the respective sky-blue and red-ochre forces of *technology-push* and *market pull* that shape their work (Fig. 1).

It remains fundamentally complex and challenging for scientists to pass the familiar white light of their academically focussed basic research through the political, economical, sociocultural, technological, legal and environmental (PESTLE) prisms of society and to convincingly connect the ‘blue’ and ‘ochre’ endpoints of the spectrum of their work in the hearts and minds of all stakeholders. The diverse range in levels of scientific understanding, skillsets and interpretations amongst these stakeholders makes for a colourful kaleidoscope of viewpoints and concerns about predicted outcomes of basic research in emerging sciences, such as synthetic biology and genome engineering. What often passes as future predictions in some of these partially informed conversations border on dubious prophecies, naïve fantasies, reckless speculations and thoughtless guesswork. In this context, the scientific method is still the most reliable predictor of future outcomes. However, the journeys of frontier scientists towards impactful discoveries are mostly along obstacle-strewn pathways with many unpredictable twists and turns.

There are no guarantees in basic science and predictable outcomes are rare. The only accurate prediction is that basic scientific research will continue to produce new breakthroughs, which will largely go unnoticed in the broader community. Even with broad media coverage, examples of recent major scientific breakthroughs that still struggle to be well understood by the public include the discovery that humans carry genes of Neanderthals and that these two species—*Homo sapiens* and *Homo neanderthalensis*—likely interbred; that there is at least one other earth-sized planet orbiting a star nearest to the sun, amid speculations about other potentially habitable planets; that the Higgs boson particle exists, thereby explaining why objects have mass; that memories of rats can be erased, restored and transformed by shining beams of coloured light on their brain cells; that gravitational waves (ripples in space-time released by a black hole collision more than a billion years ago) are real, thereby confirming Albert Einstein's prediction a hundred years ago; and that genes are rewritable with the aid of CRISPR gene-editing technology. How many of these scientific discoveries really broke through and penetrated broad-based public consciousness and understanding of the implications for a future world? Yet it is unexpected discoveries and technological advances like the acceleration of relatively inexpensive DNA-sequencing and DNA-synthesis technologies, together with the adaptation of microbial CRISPR-Cas systems for DNA-editing and genome-engineering tools, that shifted the world of biosciences from a ‘genome-read’ to a ‘genome-write’ paradigm at a blistering pace. It is for this reason that, along with other practitioners of basic science, synthetic biologists—the software engineers of life—remain so enthusiastically interested in the as-yet-unimagined discoveries to be made along the unpredictable ‘airways’ of their ‘blue-sky’ research. Their instincts, backed by ample examples from past basic scientific advances in, for example, synthetic chemistry, tell them that a string of ‘blue-sky’ breakthrough dots from their laboratories will eventually connect with ‘ochre-earth’ challenges and expectations of the general public. The well-trained eyes of seasoned scientists see many red-ochre dots embedded in every sky-blue dot they want to discover. They know that the lines of dots connecting the ‘blue-sky’ and ‘ochre-earth’ ends between ingenious science and inventive innovations are real—they just cannot always predict how many dots will be required and how straight or curly the dotted lines will run.

In the case of synthetic chemistry, when chemists of the mid-19th century took a radically different direction in their probing of structural matter by combining their traditional analytical approaches with their newly developed synthetic approaches, they opened up limitless opportunities for improving fundamental understanding of chemical compounds and novel practical applications (Yeh and Lim 2007). With their traditional analytical approaches, they could only decipher what they perceived up to that point. However, by being able to synthesise existing and new molecules—including molecules and chemical compounds that did not exist in nature—they were able to develop a much deeper understanding of the fundamental principles of chemical structure and reactivity, which in turn spawned the modern pharmaceutical and chemical industries.

**Figure 2.**
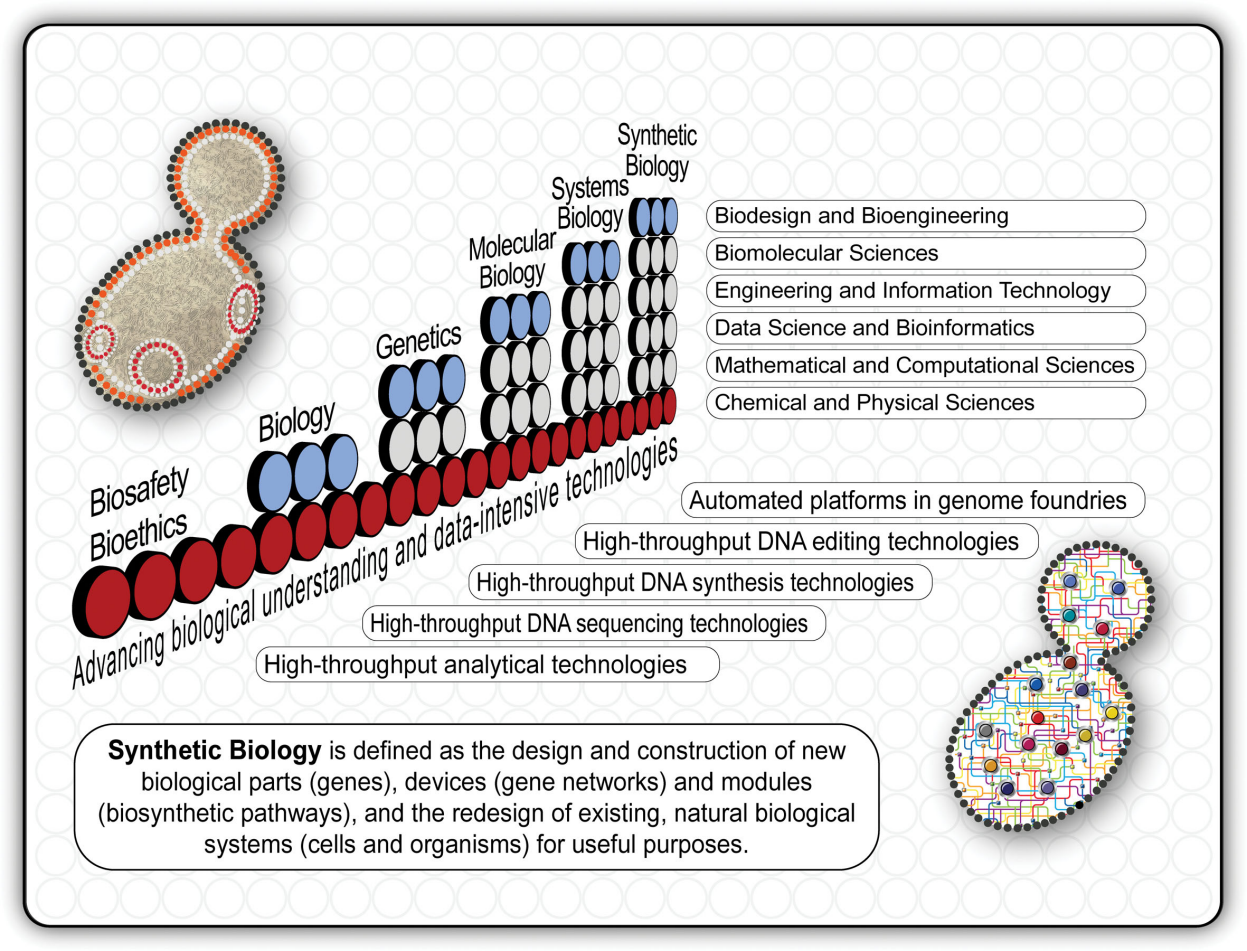
The connection between contemporary Biology and some of its many branches supported by recent advances in smart, data-intensive technologies and bioengineering tools. In classical terms, *Biology* can be defined as the study of the morphology, physiology, anatomy, behaviour, origin and distribution of living organisms. During the previous century, Biology formed several new branches, including Genetics, Molecular Biology and Systems Biology. *Genetics* entails the study of the patterns of inheritance of specific traits, relating to genes and genetic information, including studies relating to the mechanisms of hereditary transmission and the variation of inherited characteristics among similar or related organisms. With *Molecular Biology*, researchers seek to understand interactions between the various components of a cell, including interactions between the different types of deoxyribonucleic acid (DNA), ribonucleic acid (RNA) and protein biosynthesis, and to learn how these interactions are regulated. *Systems Biology* extended this ‘reductionist’ approach to a more ‘holistic’ one by using computational and mathematical modelling of complex biological systems to ‘reverse-engineer’ cellular networks. Building on the conceptual and technological advances gained from studies in Molecular and Systems Biology, this century spawned a new offshoot, *Synthetic Biology*. This emerging field combines molecular approaches with engineering principles to ‘engineer’ genetic systems by constructing collections of modular parts to design, build and fine-tune gene regulatory networks. Biosafety and bioethics are fundamental to research success in Biology and all of its branches.

Biology is now undergoing a similar transition from the late-20th century capability of deciphering the DNA sequence information of countless viral, microbial, plant, animal and human genomes to this century's capability of synthesising genome-length DNA sequences. It is a transition—recognised as the emerging discipline of synthetic biology—that demands a much deeper level of biological understanding that we presently lack. Synthetic biology is a multifaceted discipline that, amongst other things, combines advanced biomolecular and computational sciences with information technology and engineering. One definition of this emerging discipline is the design and construction of new biological parts (genes), devices (gene networks) and modules (biosynthetic pathways), and the redesign of biological systems (cells and organisms) for useful purposes (Fig. 2). Its subdiscipline of synthetic genomics seeks to design and build genomes of various types, including reinvented and recoded genomes as well as minimal genomes.

The first sign of how synthetic genomics was going to catalyse this transition during the 21st century was when a DNA copy of the entire 7458-nucleotide poliovirus’ RNA genome was synthesised in 2002 (Cello, Paul, and Wimmer 2002) and the 5386-bp genome of the bacteriophage *Phi-X174* in 2003 (Smith *et al*. 2003). Since then a few more genomes of viruses and transposons were synthesised in their entirety. However, in comparison to bacterial genomes, these synthetic viral genomes were minuscule and therefore much less expensive to synthesise than those of prokaryotes. Fortunately, as was the case with the cost and accuracy of genome sequencing, DNA synthesis technologies continued to be improved, thereby making *de novo* genome synthesis faster, cheaper and more precise: the synthesis of the first bacterial genome became possible only 5 years after the first viral genome was synthesised.

The 583-kb genome of *Mycoplasma genitalium* was chemically synthesised in full in 2008 (Gibson *et al*. 2008). This was followed by the synthesis of the 1.1-Mb chromosomal DNA of *M. mycoides* in 2010; the result was almost identical to *Mycoplasma mycoides*’ natural genome (Gibson *et al*. 2010). When this chemically synthesised circular *M. mycoides* chromosome was successfully used to replace the genome of *M. capricolum* and produced viable replicating bacterial cells, a Rubicon was crossed in bioscience. With this achievement biology irreversibly transitioned from a discipline that was (and still is) largely about observing and testing what happens when a few ‘buttons are pressed’ in the laboratory to a ‘design-build-test-learn’ approach.

Since then, a reduced (minimal) version (531 kb carrying 473 genes) of the *M. mycoides* genome (Hutchison *et al*. 2016) and a recoded genome of *Escherichia coli* (Lajoie *et al*. 2013) were synthesised as versatile platforms for further investigations into whole-genome design. The need for synthetic genomic approaches to gain higher-resolution insights into the biological complexities of living organisms than that permitted by traditional approaches became more evident when it was found that 149 of those 473 genes residing on the *M. mycoides* synthetic minimal genome were of ‘unknown function’ but deleting any one of them was lethal (Hutchison *et al*. 2016). In other words, even under tightly controlled laboratory conditions with the simplest set of synthetic genes to build ‘synthetic’ bacteria, the functionality of almost a third of the genomic content of the tiniest prokaryote remains a mystery.

This demonstrates that synthetic genomics—as a subdiscipline within the broader field of synthetic biology—is still maturing and is only now nearing a point where the function of interdependent genes might be probed in a combinatorial manner. Using such an approach to annotate the 149 genes of unknown function in the *M. mycoides* synthetic minimal genome remains a daunting task. However, without a synthetic genome for any eukaryote, such a task will be impossible in higher organisms. For yeast biologists, such a combinatorial approach would necessitate the availability of a synthetic yeast genome before the genome sequence of the world's simplest and best-studied eukaryote can be fully deciphered and its many genes of unknown function can be annotated.

### Yeast—a dot-connecting model organism with a rich history of industrial applications

Through the foggy lenses of the unacquainted, yeast is merely a unicellular fungus millions of times smaller than the dot at the end of this sentence. However, through their microscope lenses, yeast biologists see yeast cells as strings of budding dots with awesome potential. These tiny eukaryotic cells have the power to connect intriguing scientific questions with answers, solutions, breakthrough discoveries, inventions, innovations and the knowledge needed to help overcome some of the world's gravest grand challenges pertaining to health, food, water, energy, employment and the economy. In this context, *Saccharomyces cerevisiae* has, over time, emerged as the most successful dot-connecting yeast species and eukaryotic model organism—a lighthouse navigation beacon that illuminates and guides scientific ingenuity from the laboratory to inventive innovations in the field.

Fermentation is the world's oldest method of food storage and preservation, and almost every person consumes some form of fermented food regularly. For thousands of years, *Saccharomyces* has been joining the dots between humanity and fermented foods and beverages. The connection between the domestication of *Saccharomyces* and the evolution of civilisation spans seven millennia or more (extensively reviewed by Pretorius 2000; Chambers and Pretorius 2010; Jagtap *et al*. 2017). Through its rising fermentative power, this budding yeast has been brewing beer, sparkling wine and leavening bread dough since year dot. These oldest yeast-driven biotechnological processes irreversibly connected *Saccharomyces* with our dietary requirements, cultural activities, industrial development, scientific ambitions and our indomitable quest for understanding the fundamentals of life, technological advancement, social progress, economic development and modernisation (recently reviewed in different contexts by Pretorius 2016; Goold *et al*. 2017; Pretorius 2017a,b).

A continuation of connecting the dots between fundamental understanding of biological systems and human futures (improved quality of life in the context of a sustainable environment) will require the revelation and contextualisation of more dots, i.e. ideas, data points, scientific evidence and proof of hypotheses. Put differently, unlike the simplicity of using a pencil to connect the numbered dots in a child's colouring book to reveal the ‘Big Picture’, it is much more complex to join the dots in biological systems where not all the dots are numbered or even known to exist. In a eukaryote, the ‘connect-the-dots’ picture often looks more like a random-dot stereogram. It is therefore understandable that researchers seek to uncover the molecular intricacies of a single yeast cell before they attempt to do the same in higher eukaryotes, such as plants, animals and humans—higher eukaryotes consist of trillions of cells and the number of cell types in an organism is not even accurately known, with new cell types discovered daily by single-cell sequencing.

Over time, *S. cerevisiae* developed into an ideal food-grade eukaryotic model organism for academic studies as well as industrial applications beyond the traditional fermentation-based industries of baking, brewing, winemaking and biofuel production. The characteristics that make this yeast such a broad-shouldered study model include its relatively short reproduction time (90 min under optimal growth conditions); simple and inexpensive cultivation as stable haploid, diploid and polyploid cells in defined media; efficiency of sporulation and cross-hybridisation between two stable opposite mating types (**a** and **α**); ease of mutant isolation and mapping; efficacy of genetic transformation, maintenance of multiple copies of circular plasmids as well as chromosomal integration through homologous recombination; rare pathogenicity; relatively small genome size [∼12 Mb (non-redundant) to ∼14 Mb (total) genome carrying ∼6000 genes on 16 chromosomes varying in length from ∼200 to ∼2000 kb]; and availability of chip-based gene deletion libraries (Pretorius 2017a). Not only was this yeast the first microorganism to be domesticated for the production of fermented foods and beverages in ancient times, it was also the first microbe to be observed under the microscope, by Antonie van Leeuwenhoek in the late 1600s, and described as a living biochemical agent of transformation by Louis Pasteur 200 years later. In more recent times, *S. cerevisiae* was the host for the manufacturing of the first genetically modified (GM) vaccine (against hepatitis B) and the first GM food enzyme (the milk coagulation enzyme, chymosin, for cheese making). In 1996, a haploid laboratory strain (S288c) of *S. cerevisiae* became the first eukaryote whose genome was fully sequenced (Goffeau, Barrell and Bussey 1996; Oliver 1996).

Over the years, researchers have been stacking data point atop data point about *S. cerevisiae*’s genome, transcriptome, proteome and metabolome, and have been layering nano-insight on nano-insight about its fluxome, interactome and epigenome. Today, researchers can stand back from the pixelated pictures of past discoveries in biomolecular science and allow the unintelligible constellation of dots to form a clearer image of the yeast cell system. However, it is not enough to have access to more and more terabytes of data points (i.e. dots) in yeast systems biology; it is the diversity of expertise from other disciplines, such as chemical, physical, mathematical and computational sciences alongside cutting-edge developments in information technology and engineering, which is now required to accelerate the connecting of dots with the aid of synthetic biology technologies.

Once again, *S. cerevisiae* is showing dot-connecting ‘technological leadership’ amongst eukaryotes by gaining ‘first-mover advantage’ in synthetic genomics. Following the synthesis of the first viral and bacterial genomes during the ‘noughties’, a large international project—the *Synthetic Yeast Genome* project (known as the *Yeast 2.0* or *Sc2.0* project)—is now underway to produce the world's first functional synthetic eukaryotic genome by the end of 2018 or soon thereafter (see the Sc2.0 website www.syntheticyeast.org).

The Sc2.0 project's success lays within its clarity of purpose supported by audacious goals and evolving breakthrough technologies in synthetic biology; its unique collaborative approach within a tightly connected, multidisciplinary partnership guided by a sound self-regulation and self-governance structure; and its shared, unambiguous stance on biosafety, bioethics and ambition to advance and apply scientific knowledge for the benefit of humanity in a planetary context. In this article, the dots are being connected between the Sc2.0 project and the future of synthetic genomics in the era of ‘Biotech 2.0’—a bioeconomy comprising industry-based biodesign, bioengineering, biomanufacturing and biorobotics, and expected to boom in the years to come.

## THE CONNECTION BETWEEN YEAST GENOME DESIGN AND GENOME ENGINEERING

### Connecting audacious biodesign goals to enabling technologies and methodologies

The Sc2.0 project is an ambitious, large-scale ‘blue-sky’ project. It is driven by the same degree of inquisitiveness, intuitions and aspirations for discovery, breakthroughs and application of new knowledge as described above. The concept of building a synthetic version of a reinvented yeast genome is based on the premise that to fully understand what makes an organism tick, one should be able to design and redesign one. This concept was first floated in the mid-2000s but only took shape at the start of this decade (Pennisi 2014). The Sc2.0 project aims to design and build a rewritten yeast genome from scratch so that we can test and extend the limits of the current body of biological knowledge.

This project builds on a well-researched laboratory strain of *S. cerevisiae*, S288c, which has been undergoing laboratory propagation since its isolation from a rotting fig in Merced, California, in 1938. The Sc2.0 experimental work is conducted with the ‘BY’ lineage, directly derived from the laboratory-adapted S288c parental strain. These heterothallic haploid strains carry auxotrophic mutations, making them dependent on specific laboratory-supplied nutrients in their growth media. These strains have also lost the ability to ‘forage’ for nutrients through structures called pseudohyphae, thereby rendering them uncompetitive against prototrophic homothallic yeasts in the wild and making them a safe option for laboratory experimentation. Their reduced fitness in the wild together with the wealth of data and the availability of the full genome sequence of S288c made these food-grade BY strains with GRAS (generally regarded as safe) status an obvious choice for fulfilling our vision of synthesising a designer eukaryotic genome without significant biosafety-related risks.

The overarching purpose of the Sc2.0 project is to design and chemically synthesise a slightly modified version of the S288c yeast genome. Our goals are to use this man-made version of *S. cerevisiae*’s genome to answer a wide variety of profound questions about fundamental properties of chromosomes, genome organisation, gene content, function of RNA splicing, the extent to which small RNAs play a role in yeast biology, the distinction between prokaryotes and eukaryotes, and questions relating to genome structure and evolution while recognising that the eventual ‘synthetic yeast’ being designed and refined could ultimately play an important practical role (see www.syntheticyeast.org). It is also hoped that this project will, amongst other things, accelerate the annotation of *S. cerevisiae*’s many genes of unknown function—still an unfinished task, more than 20 years after the announcement of its genome sequence.

**Figure 3.**
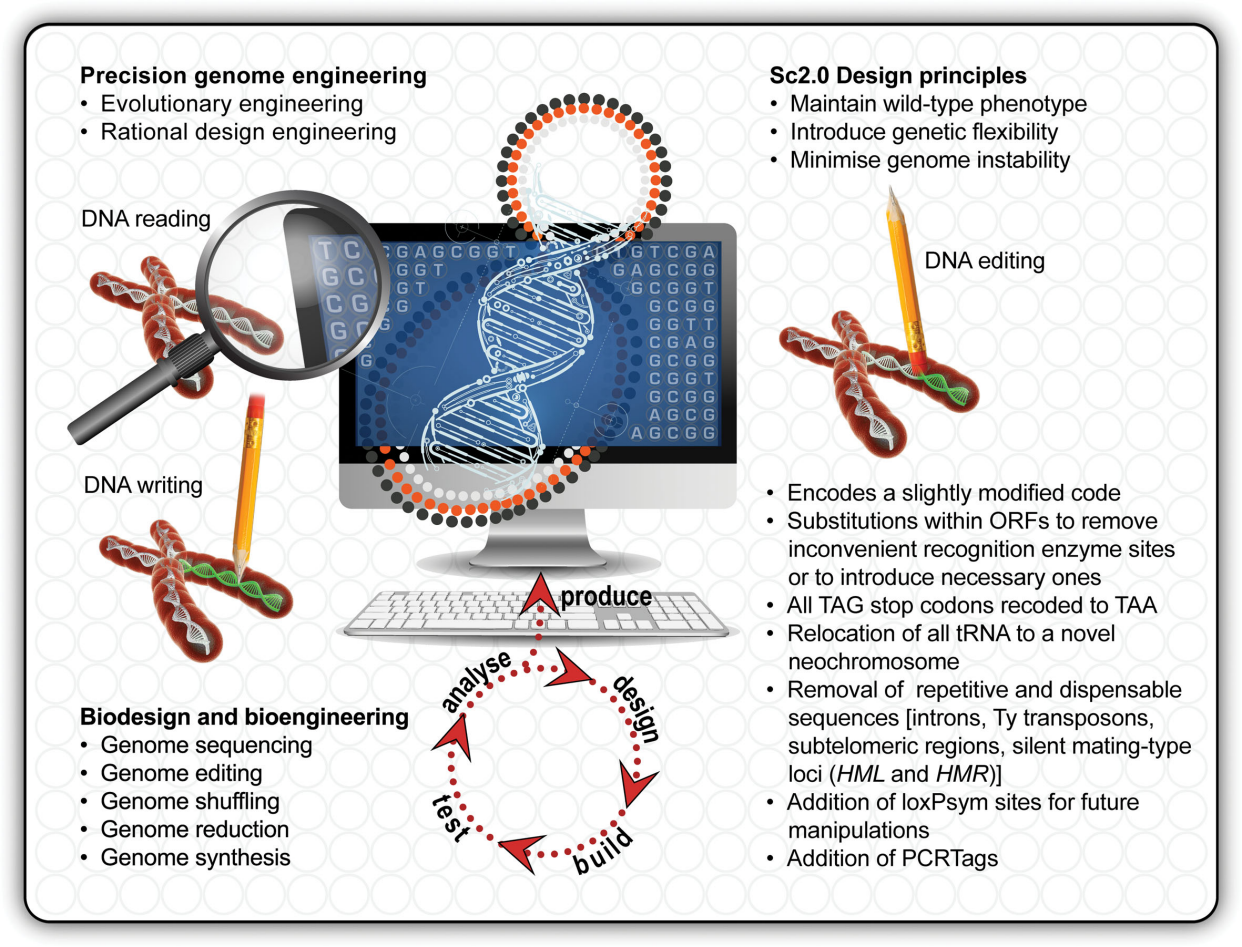
The connection between biodesign and bioengineering. Precision genome engineering connects evolutionary and rational design engineering by joining the dots between genome sequencing (*DNA reading*), genome synthesis (*DNA writing*), genome shuffling/scrambling, genome reduction/recoding and CRISPR editing (*DNA editing*) on one side and ‘design-build-test-analyse-produce’ capabilities on the other. The principles guiding the design of a reinvented Sc2.0 genome balance a desire to preserve the wild-type phenotype of *Saccharomyces cerevisiae* S288c while introducing flexibility and minimising sources of genomic instability resulting from the repetitive nature of native DNA sequences.

To answer these probing questions about the fundamental inner workings of a yeast cell's genetic make-up, the blueprint for a synthetic genome had to be designed, curated, streamlined and reorganised to encode a slightly modified genetic code. To do that, an optimal genome design framework had to be developed that would allow the design team to make coordinated modifications to DNA sequences at both the base-pair level and genome scale (Fig. 3). Therefore, the BioStudio software program was specifically developed as an open-source framework for eukaryotic genome design by a team at Johns Hopkins, led by Joel Bader, to reinvent the blueprint for the designer Sc2.0 genome, which included changes that can be tracked and rolled back at multiple scales (Richardson *et al*. 2017).

The aim of the design principles was to simplify the assembly of the synthetic chromosomes. Specific base substitutions within some of the open reading frames (ORFs) are included in the redesigned genome to incorporate necessary enzyme recognition sites or eliminate inconvenient enzyme recognition sites. Along with these changes, recognisable PCRtags—short recoded sequences within certain ORFs facilitating a polymerase chain reaction (PCR)-based assay—are also included in the design so that the synthetic DNA can be differentiated from native DNA. Other notable variations in the design include the addition of many loxPsym sites for future genome scrambling purposes; all TAG stop codons are recoded to TAA; all repetitive and dispensable sequences, such as the five families Ty retrotransposons [a total of ∼50 copies bounded by long terminal repeat (LTR) sequences], pre-tRNA and pre-mRNA introns, subtelomeric regions and silent *HML* and *HMR* mating-type loci (located on Chromosome 3) are omitted; and all tRNA genes are relocated to a novel neochromosome (Richardson *et al*. 2017). The decision for deleting the retrotransposons and their LTRs from the design was to remove as much dispersed repetitive DNA as possible from the genome, thereby potentially delivering a more stable synthetic genome free of mobile elements. The pre-mRNA introns were precisely deleted from the design, excepting (for now) those genes with evidence of fitness defect caused by intron omission. The *HAC1* intron, which uses separate splicing machinery and is known to play a critical role in the unfolded protein response, was kept in the design. The rationale for the relocation of all tRNA genes to a specialised neochromosome encoding only tRNA molecules was based on the fact that tRNAs lead to genome instability by replication fork collapse—presumably due to collision with tRNA polymerase PolIII (Richardson *et al*. 2017).

It is anticipated that these rather conservative designer changes will not cause any fitness defects but will make allowance for future genome manipulations and further investigations. For example, the site-specific recombination sequences at carefully-chosen positions in the synthetic chromosomes are not expected to impact fitness upfront. The process, however, will allow *in vitro* evolution or SCRaMBLE of the transformed yeast cells to reveal which DNA sequences are, in fact, dispensable when the appropriate recombinase is transiently expressed and survivors are identified.

The methodology to build the designer Sc2.0 genome is based on a hierarchical assembly plan (known as ‘switching auxotrophies progressively for integration’ or ‘SwAP-In’) specifically devised for this ambitious project (Richardson *et al*. 2017). One approach is to assemble building blocks of ∼750 bp into minichunks of ∼3 kb which, in turn, are assembled into chunks of ∼10 kb before they are assembled into 30–60 kb megachunks. Alternatively, the synthesis of 10 kb chunks can be outsourced and then assembled into 30–60 kb mega-chunks. So typically, three to six 10 kb chunks or 30–60 kb megachunks of synthetic DNA can each be integrated one by one into the yeast genome. The termini of each synthetic 10 kb chunk are designed to encode specific restriction enzyme sites that enable directional assembly into 30–60 kb megachunks by *in vitro* ligation. The rightmost terminus of every right-end chunk (from left to right) is also designed to contain a selectable marker (e.g. *URA3*). The 30–60 kb megachunks can then be transformed into the auxotrophic haploid yeast cells. In the transformants, the corresponding native sequence is replaced through the mechanism of *in vivo* homologous recombination while the embedded selectable marker enables the growth and identification of yeast cells that underwent integrative transformation. The integration of the leftmost megachunk overwrites a kanMX cassette, previously introduced into the native chromosome sequence for negative selection purposes. As each subsequent megachunk is incorporated, the auxotrophic marker used in the prior round is eliminated by recombination and selection is imposed for a second selectable marker (e.g. *LEU2*). This strategy allows for the remainder of the synthetic chromosome to be assembled *in vivo* with alternating selection for *URA3* and *LEU2* markers. In addition to monitoring the loss and gain of auxotrophic markers, integration of synthetic DNA and loss of native sequences were confirmed by the presence of the designed PCRtags. These PCRtags are designed as synonymously recoded sequences that permit selective amplification of synthetic or native DNA, which serve as identifiable ‘watermarks’ for synthetic DNA throughout all synthetic chromosomes.

Since all transformed strains are haploids, their phenotypes (such as fitness to grow on appropriate selective and non-selective culture media) can be easily monitored after each cycle of incorporating a synthetic DNA segment (Richardson *et al*. 2017). A relatively sensitive and efficient way to detect major fitness defects in strains carrying synthetic DNA sequences is by continuously comparing the colony size of transformants with that of the parental strain plated out on appropriate selective and non-selective culture media. Routine monitoring of growth on glycerol as carbon source at 37˚C reveals a wide variety of ‘bugs’ also known as changes in fitness, because this growth regimen represents a doubly stressful condition, with a requirement for full-on mitochondrial function as well as thermotolerance. In addition, after about 10 cycles of incorporating synthetic megachunks (∼300–500 kb), transcript profiling is used to determine whether the inclusion of synthetic DNA segments caused any changes in overall gene expression. In the event that fitness defects are detected, DNA sequencing and a systematic approach to ‘debugging’ the synthetic sequences are undertaken until the cause of the defect is identified and rectified.

Once the design for the synthesis of the 16 chromosomes, the strategy and the methodology were in place, the daunting task of synthesising the entire genome—chromosome by chromosome from the bottom up—could commence in all earnest. The next step was to pull an international consortium of research teams together—teams led by committed and collaborative researcher leaders, each with the required expertise and resources to build their assigned chromosome(s).

### Connecting a multidisciplinary network of international partners and collaborators

Great discoveries and technological improvements invariably involve the cooperation of many minds. Breakthroughs in our understanding of scientific fundamentals and innovations are rarely the result of one person's endeavour. When the Sc2.0 project was conceived, a modern approach termed ‘committed collaboration’ was adopted. This approach requires an exchange of ideas in a spirit of mutual trust. It is a collaborative approach that has become more evident in contemporary, multidisciplinary research focussed on solving the most important issues facing the world.

But what does ‘committed collaboration’ actually mean for Sc2.0 researchers seeking to understand the ‘fundamentals’ in the context of potential applications to today's grand challenges? Collaboration, teamwork, partnerships and networks are all buzzwords that are often used but seldom thoughtfully understood. Several questions were discussed as the Sc2.0 consortium was being established. For example, if we were going to tackle an ambitious project such as designing and building the world's first synthetic eukaryotic genome and turn the emerging science of synthetic biology to our advantage, how could we best collaborate to succeed? How could we avoid a swathe of ‘memoranda of understanding’ between research agencies around international partnerships becoming an end in themselves? How could we ensure that top-down, centrally invoked research partnerships did not develop into camouflages in which togetherness becomes a disguise for mediocrity? What could we, as a group, put in place to help research leaders, eager to tear down silos, to remember that the goal is not *collaboration* itself, but *results*? We knew that while collaborative efforts can achieve spectacular synergies, many boomerang—wasting time, money and resources. The Sc2.0 project needed contemporary research leaders who understood that when collaborations are activated for the right reasons and managed well, they optimise resources, speed up success and create a positive experience for all concerned.

**Figure 4.**
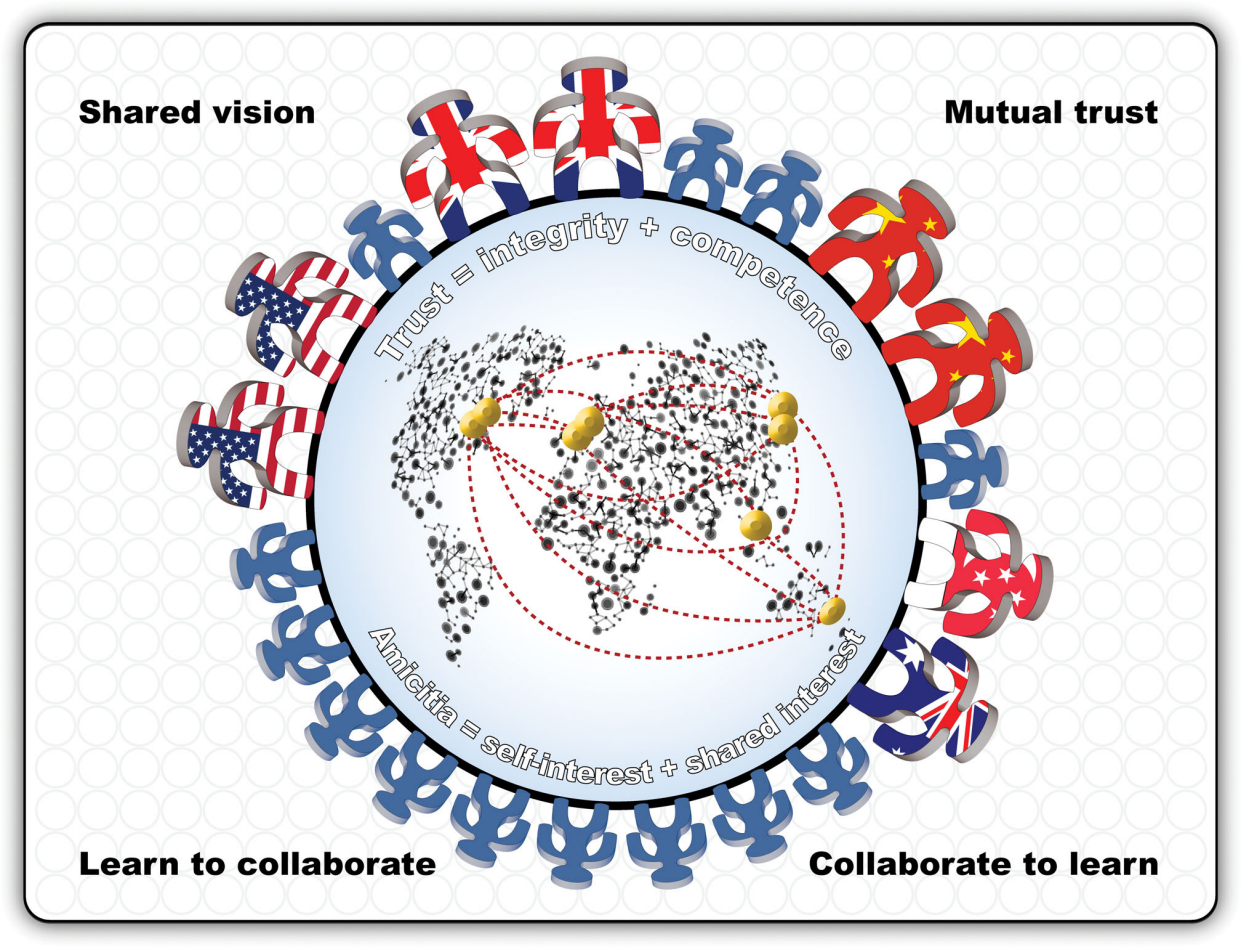
A connected alliance of global partners committed to designing and building of the world's first synthetic yeast genome. This multidisciplinary collaboration across geopolitical boundaries is based on the ‘amicitia’ principle of balancing shared interests with self-interest. This type of ‘committed collaboration’ is underpinned by mutual respect and trust amongst the Yeast 2.0 partners. In this trust relationship, trust is defined by the integrity and competence of the participating chromosome-building teams.

A common theme in the Sc2.0 partnership has always been mutual trust coupled with effective communication. Whether the researchers worked independently, or as a group, effective communication networks were required to inspire and inform all parties (Fig. 4). Under the leadership of Jef Boeke (New York University), the leaders of the other Sc2.0 chromosome-building teams, in the USA (Srinivasan Chandrasegaran from Johns Hopkins University), UK (Tom Ellis from Imperial College London; Patrick Yizhi Cai formerly from the University of Edinburgh and now at Manchester University), China (Yingjin Yuan from Tianjin University; Junbiao Dai formerly from Tsinghua University now at Shenzhen Institute of Advanced Technology; Yue Shen from BGI), Singapore (Matthew Wook Chang from the National University of Singapore) and Australia (Sakkie Pretorius from Macquarie University and supported by Daniel Johnson from The Australian Wine Research Institute), committed to building trust and sharing ideas within the Sc2.0 consortium.

This partnership has grown tighter over the past seven years—akin to the concept of ‘amicitia’—a word borrowed from ancient Rome. In the spirit of ‘amicitia’, collaborative action is performed in an environment of mutual respect balanced with self-interest: one commits to collaborate and to contribute, but one is never expected to experience harm or to neglect one's own self-interest. For us in the Sc2.0 alliance, the principle of ‘amicitia’ provides a useful guideline. It means that all the Sc2.0 researchers, partners, collaborators and other stakeholders are expected to protect their own interests. However, it also means that we look out for each other and help one another so that we can achieve our shared goals. Within the Sc2.0 consortium, we are learning to collaborate and we are collaborating to learn. We are committed to the principle of co-creation and co-training of ‘next-generation’ researchers and the future workforce. Over geopolitical and disciplinary boundaries, we are exploring new ways to work with each other to find new ways forward in our resolve to replace *S. cerevisiae*’s 16 native chromosomes with 16 chemically synthesised chromosomes. We are in this together, and together in the spirit of ‘amicitia’, we are committed to deliver the world's first synthetic eukaryotic genome in the foreseeable future.

The spirit of ‘amicitia’ within this close-knit Sc2.0 alliance also provides the freedom to pursue a secondary objective, namely to join the dots across the skyline between the ‘blue-sky’ learnings of the shared core Sc2.0 project and the ‘ochre-earth’ aspirations of each of the collaborating partners’ individual applied ‘side-projects’. In other words, when taken together, the Sc2.0 members’ research agendas are directed towards increasing fundamental understanding of the inner mechanics of yeast cells in a context responsive to end-users’ applied needs at levels of both problem selection and experimental design. Looking at the far horizons of the rapid developments in synthetic biology, our ‘amicitia’ approach serves as a connecting boundary line between the ‘blue-skies’ of our ‘pure’ research and our craving for ‘ochre-earth’ applications of our research for the benefit of people and planet.

### Connecting scientific freedom and social responsibility

Rapid advances in emerging scientific fields sometimes outpace the capacity of governments to legislate for appropriate regulatory coverage of fast-developing technologies. New and unanticipated issues often arise as a new research field develops while initial concerns dwindle as more data, insight and experience are gained. It is therefore incumbent on researchers involved in such a young and dynamic field to be continuously mindful of the potential implications of their work on safety, ethics and policy in the context of the scientific and technological challenges being overcome. In the absence of new or updated regulatory frameworks for developing and potentially controversial sciences, such as synthetic biology, self-governance and self-regulation are vital to the success of pioneering research (Sliva *et al*. 2015).

Synthetic genome engineering is a continuum of genetic engineering. Like the pioneers of recombinant DNA technology and genetic engineering back in the 1970s, today's synthetic biologists are also facing the challenge where, technically, almost anything seems possible. In this rapidly developing and dynamic field of synthetic genomics, the excitement about seemingly limitless possibilities, benefits and rewards is sky-high but so are some stakeholders’ anxiety levels and concerns about potential risks relating to both ‘bio-terror’ and ‘bio-error’. The potential for bioterrorism is well understood and it is the responsibility of researchers to ensure that every effort is made to combat the risk of nefarious intent. As for unintended ‘bio-errors’, concerns largely revolve around experiments aimed at the production of novel bioengineered organisms for environmental release or use in medicine and food, and so-called ‘dual-use’ experiments whose products are intended to benefit society but also hold the potential to cause harm (Sliva *et al*. 2015). Thus, the bright and dark sides of synthetic biology's potential contribute to the field's mythos among the general public as sources of both fascination and apprehension. Therefore, pioneering synthetic biologists bear a significant responsibility to ensure that the experiments they are contemplating or performing are conducted in a way that maximises the bright side of opportunity for benefit while minimising the dark side of risk for harm. They also bear the responsibility for continuous civic engagement by communicating effectively with social scientists and maintaining an open, meaningful dialogue with the public about the biosafety, bioethical and governance aspects of their work.

**Figure 5.**
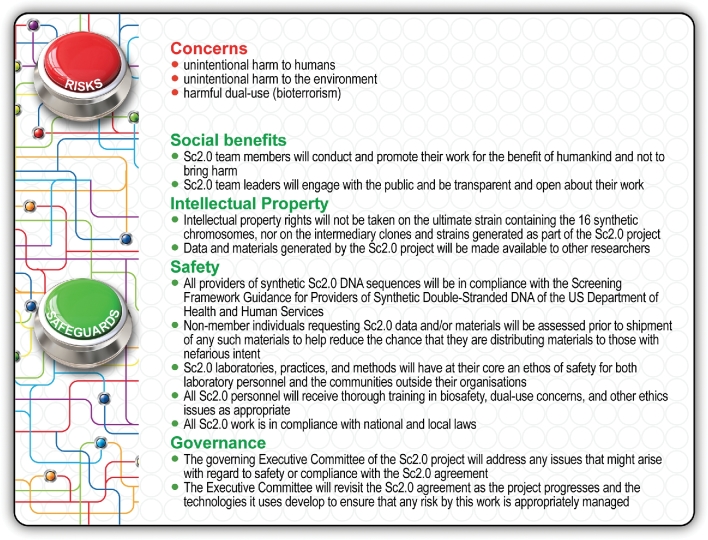
The connection between scientific freedom and social responsibility in the Yeast 2.0 collaboration is balanced by a project-level agreement and guided by a statement on safety and ethics (Sliva *et al*. 2015). This approach of ongoing oversight, self-regulation and self-governance provides the Sc2.0 consortium with an effective and dynamic framework for maximising the benefits of this large-scale project and minimising the risk for harm or damage.

From the outset, the Sc2.0 consortium members embraced their social responsibilities and adopted a truly multidisciplinary approach and proactive attitude by collaborating with social scientists and engaging with the public and regulatory authorities. Also, because this international consortium comprises researchers from different disciplines, cultural backgrounds, geopolitical jurisdictions and diverse settings, it was vital to develop and officially agree upfront on a common set of principles to guide this large-scale project. In the spirit of self-regulation and self-governance, every partner organisation involved in the Sc2.0 project had to adopt a legally binding agreement, stipulating that each individual researcher working across the various nodes will strictly adhere to the principles outlined in a published statement on safety, ethics and governance (Fig. 5). We all had to sign on the dotted line. The Sc2.0 project-level agreement and statement on safety, ethics and governance address the core issues relating to societal benefits, intellectual property, safety and governance of this international venture (Sliva *et al*. 2015).

Every member of the Sc2.0 consortium committed upfront to conduct and promote their research agendas for the benefit of people and planet, and not to cause any harm or damage (Sliva *et al*. 2015). All team members agreed to engage with the public on an ongoing basis and to be totally transparent about their experimental work. In accordance with the agreed principles, intellectual property rights will not be claimed on the ultimate strain containing the 16 synthetic chromosomes, nor on the intermediary clones and strains generated as part of the Sc2.0 project. Also, materials and data generated by this project will be accessible to other researchers on the proviso that non-member parties requesting Sc2.0 data and/or materials are willing to be assessed prior to shipment of any such materials, to minimise the risk of distributing materials to those with nefarious intent. It was agreed that all providers of synthetic Sc2.0 DNA fragments will have to comply with the *Screening Framework Guidance for Providers of Synthetic Double-Stranded DNA* of the US Department of Health and Human Services. The principal leaders of each of the Sc2.0 nodes gave the assurance that their research facilities, practices and methods have at their core an ethos of safety for both laboratory personnel and the communities outside their institutions. Before commencement of laboratory work, every individual working on the Sc2.0 project would be trained in biosafety, dual-use concerns and other ethics issues as appropriate. Given that the various Sc2.0 teams operate in diverse geopolitical jurisdictions, all Sc2.0 work must, of course, also comply with relevant national and local laws. A governing executive committee oversees the entire Sc2.0 project. This committee addresses any compliance issues as they arise and revisits relevant aspects of the project-level agreement as the research progresses and technologies develop to ensure that any risk is appropriately managed according to the *NIH Guidelines for Research Involving Recombinant or Synthetic Nucleic Acid Molecules*. These guidelines are periodically reviewed and revised to keep pace with current synthetic biology research. If any revisions are made to these guidelines that are relevant to Sc2.0 research, the project-level agreement will be reassessed, updated where appropriate and implemented accordingly. This approach of ongoing oversight, self-regulation and self-governance according to current legislation and widely adopted guidelines provide the Sc2.0 consortium with an effective and dynamic framework for maximising the benefits of this large-scale project and minimising the risk for harm or damage (Sliva *et al*. 2015).

Annual *International Yeast 2.0 and Synthetic Genomes* conferences are being held to, amongst other things, network, share ideas, monitor progress and strengthen collaboration. So far, six conferences have been staged in Beijing (17 April 2012), London (12 July 2013), Taormina (20 June 2014), New York (16–17 July 2015), Edinburgh (8–9 July 2016) and Singapore (13–16 June 2017). These conferences contributed to the connectedness within the Sc2.0 consortium and the successful synthesis of over one-third of the chromosomes. Currently, the Sc2.0 ‘yeast whisperers’ are working feverishly to complete the synthesis of the remaining chromosomes in time for the seventh—and perhaps especially celebratory—conference scheduled to take place in Sydney (26–28 November 2018).

## SYNTHETIC YEAST DOTS CONNECTED TO DATE

### Six connected Sc2.0 dots and counting

**Figure 6.**
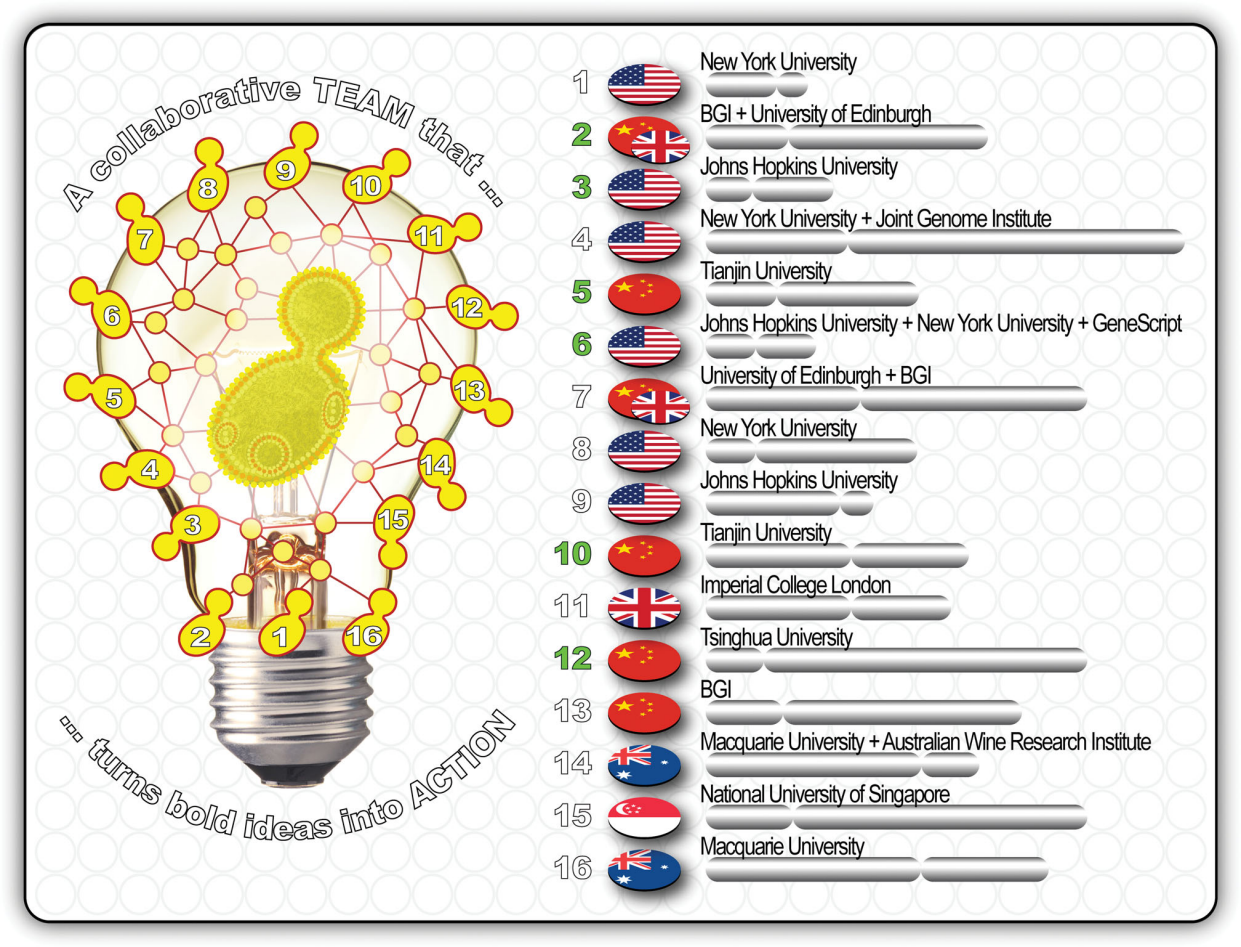
The connection between a bold idea of building the world's first synthetic yeast genome and delivering 16 synthetic chromosomes by the end of this year. A global alliance of a dozen teams from five countries is turning the Yeast 2.0 idea into action. To date, six chromosomes (depicted by green numbers) have been synthesised and swapped out for their native counterparts in *Saccharomyces cerevisiae* S288c. The full-length synthesis of the 10 remaining chromosomes is almost complete. Most teams are in the process of identifying the causes of fitness defects and debugging these imperfections.

The significant achievements of the Sc2.0 consortium to date include the smart design of the synthetic genome along with the successful SwAP-In replacement of six of yeast's native chromosomes II (Shen *et al*. 2017), III (Annaluru *et al*. 2014), V (Xie *et al*. 2017), VI (Mitchell *et al*. 2017), X (Wu *et al*. 2017) and XII (Zhang *et al*. 2017) with synthetic versions, containing important modifications (Fig. 6). As specified by the BioStudio genome design program, these *in silico* edits to the genome sequence of *S. cerevisiae* S288c entailed ∼8% reduction in overall genome size, with 1.1 Mb of the synthetic genome deleted, inserted or altered (Richardson *et al*. 2017). This design achieved the aim to maintain the ‘wild-type’ phenotype S288c as best as possible while introducing inducible genetic flexibility and minimising sources of genomic instability resulting from the repetitive nature of *S. cerevisiae*’s native DNA. To date, the Sc2.0 design principles have been thoroughly put to the test with the full-length synthesis of six out of the 16 chromosomes.

#### Synthetic chromosomes synIXR and synIII

The first concrete progress towards the building of a designer yeast genome became evident with the successful construction of a manually designed circular synthetic version of the right arm of chromosome IX (chrIXR), which was labelled synIXR (Dymond *et al*. 2011). This pioneering study demonstrated that all of the design changes later applied to the rest of the Sc2.0 chromosomes were sound, and also demonstrated for the first time that SCRaMBLE could work. A follow-up study showed that SCRaMBLE was highly random in terms of the likelihood that any two pairs of loxPsym sites would recombine (Shen *et al*. 2016). These studies paved the way for the synthesis of the first full-length yeast chromosome, i.e. the synthetic version of chrIII in 2014 (Annaluru *et al*. 2014). Coincidentally, chrIII, which carries the mating-type loci, was also the first yeast chromosome to have been fully sequenced in 1992 (Oliver *et al*. 1992). It is *S. cerevisiae*’s third smallest chromosome, covering about 3% of the yeast genome. The 316 617 bp native chrIII of the wild-type strain was successfully swapped with a 272 871 bp synthetic version synIII (Annaluru *et al*. 2014). This *in silico* edited synIII chromosome was 14% shorter than chrIII and contained over 50 000 sequence modifications, including redesigned telomeres, 98 added loxPsym sites and all TAG stop codons recoded to TAA stop codons while 11 tRNA genes, all introns, transposable elements and the silent *HML* and *HMR* mating-type loci were removed (Annaluru *et al*. 2014; Gibson and Venter 2014). It was instructive to learn that none of these sequence alterations caused any significant fitness reduction in the synIII-carrying semisynthetic strain. The results achieved with synIXR and synIII were positive signs for the full-length synthesis of the other 15 chromosomes.

Last year, five additional *S. cerevisiae* chromosomes (chrII, chrV, chrVI, chrX and chrXII) were individually swapped out for their synthetic counterparts (synII, synV, synVI, synX and synXII) in discrete strains (Mitchell *et al*. 2017; Shen *et al*. 2017; Wu *et al*. 2017; Xie *et al*. 2017; Zhang *et al*. 2017). This means that 30% of the native yeast genome has been replaced across five strains. In addition to the learnings gained previously from the ‘build-to-understand’ synIII-containing strain, several new insights came to the fore during the ‘design-build-test-debug’ cycles of the latest tranche of synthetic chromosomes.

#### Synthetic chromosome synVI

Chromosome VI was the second full-length Sc2.0 chromosome—designed to specifications—to be swapped out for a 242 745-bp synthetic version thereof (Mitchell *et al*. 2017). The synVI version is 11.3% shorter than chrVI. In synVI, >9000 bp were recoded, and 10 tRNA genes and five spliceosomal introns were deleted. However, a single non-spliceosomal intron [encoded by *HAC1* (*YFLO31W*)] was retained because of its critical role in the regulation of the unfolded response. Phenotypic, transcriptomic and proteomic analyses revealed that three unexpected phenotypic differences resulted from the edits made in the designer chromosome.

The first observation entailed partial silencing of terminal genes positioned subtelomerically relative to their locations on chrVI. Two genes adjacent to a terminal universal telomere cap (UTC)—*YFLO55W* (encoding a low-affinity amino acid permease, Agp3) and *YFR055W* (encoding Irc7 β-lyase involved in thiol production)—were downregulated in the synVI-carrying strain. This indicated that the BioStudio-specified consensus core X elements of the UTC in the Sc2.0 genome are insufficient to fully insulate telomere-proximal promoters from a so-called telomere position effect.

Second, a mitochondrial defect in the synVI-containing strain resulted in a translational impairment. This defect was attributed to a fortuitous RNA secondary structure resulting from recoding deep within the *PRE4* (*YFR050C*) gene, which encodes an essential proteasome subunit.

The third defect that needed to be restored in synVI was caused by the deletion of an upstream tRNA gene and loxPsym site insertion, which resulted in a transcriptional phenotype associated with activation of a cryptic start site and/or promoter interference in *HIS2*. Once all of the fitness-reducing sequence variations in synVI were debugged, no major growth defects were detected.

#### Synthetic chromosome synII

The modular Sc2.0 construction approach to progressively and individually swap the wild-type chromosomes chunk by chunk with their designer counterparts in discrete BY4741 and BY4742 strains was also successfully applied to the other completed synthetic chromosomes. With synII, 33 deletions, 269 insertions and 14 949 single-nucleotide substitutions were made to the sequence of chrII (Shen *et al*. 2017). The resulting 770 035 bp synII is 5.3% shorter than its native chrII counterpart. Out of the 30 introns within protein-encoding genes, 22 were removed. The eight remaining introns retained in synII were either known to cause fitness defects when deleted or they are embedded within ribosomal protein genes and omission of such introns might result in certain fitness defects.

When a strain carrying synII was subject to extensive phenotypic and trans-omics analyses, a slight but potentially significant upregulation of translational machinery was observed (Shen *et al*. 2017). This change was largely due to the deletion of 13 tRNA genes from synII; however, by restoring the tRNA copy number this variation was reversed. A growth defect in a culture medium containing glycerol as the sole carbon source at an incubation temperature of 37°C was caused by a PCRtag in *S. cerevisiae*’s *TSC10* gene, which, in turn, was responsible for misregulation of the high-osmolarity glycerol response pathway. Complementation assays and SCRaMBLE were harnessed to pinpoint the cause of this growth defect, which was then ‘debugged’ by replacing that PCRtag-distorted sequence with the wild-type version of *TSC10*. When the strain carrying the debugged synII was further characterised by phenomics, transcriptomics, proteomics, chromosome segregation and replication analyses, it was found that synII segregated, replicated and functioned in a similar fashion compared to its wild-type counterpart (Shen *et al*. 2017).

#### Synthetic chromosome synV

The design of the 536 024 bp synV synthetic version of chrV specified the deletion of two subtelomeric regions, 20 tRNA genes, 30 Ty transposons and 10 introns; the insertion of 176 loxPsym sites; 62 TAG/TAA stop-codon swaps; and 339 synonymous recordings to introduce PCRtags (Xie *et al*. 2017). No differences were detected between the strains carrying chrV and synV, except for the expression levels of five genes, *MCM3*, *YER187W*, *YER188W* and two subtelomeric genes. *MCM3* is an essential gene that encodes a subunit of the replicative helicase. However, these modest differences in expression levels did not seem to negatively impact the growth of the synV-carrying strain under the conditions tested.

As an interesting variation, synV was circularised by deleting both telomeres without altering the synthetic chromosome's gene content. The rationale for creating such a ring derivative of the synthetic chromosome was to explore whether it would be possible to develop a process that could eventually be used for modelling ring-chromosome induced disorders in human epilepsy, intellectual delay and dysmorphic features, such as leukemia and microcephaly (Xie *et al*. 2017). In this exploratory work, it was demonstrated that the circular version of synV was fully functional in yeast, except for displaying lower spore viability during meiosis. This is clearly a promising area for further investigation of genomic rearrangements, ring-chromosome evolution and human ring-chromosome disorders.

#### Synthetic chromosome synX

Designer modifications in synX—a 707 459 bp synthetic version of chrX—include the deletions of subtelomeric repeats, retrotransposons and introns, the recoding of all TAG stop codons to TAA, and the insertion of 245 loxPsym sites in the 3΄ UTRs of non-essential genes and 490 pairs of synonymous sequences in ORFs or PCRtags (Wu *et al*. 2017). Apart from these sequence alterations in synX, 24 tRNA genes were deleted and a single-copy tRNA gene, *tR(CCU)J* was relocated to the *HO* locus (responsible for homothallism in yeast). During the assembly of synX, an efficient high-throughput mapping strategy called ‘pooled PCRtag mapping’ (PoPM) was developed to identify and eliminate a couple of fitness-reducing sequence variations (bugs) from the BioStudio-designed sequence, as well as other fitness-related bugs (Wu *et al*. 2017).

First, a loxPsym site in the 3΄ UTR of a so-called ‘dubious ORF’, *YJR120W*, was found to disrupt the promoter region of an adjacent gene, *ATP2*, which encodes a subunit of the mitochondrial F1F0 ATP synthase. The deletion of this dubious ORF produced a transcriptional hypomorph of *ATP2*—a situation referred to as an ‘off-by-one error’ or ‘neighbouring gene effect’. By correcting the designed sequence and assembling a revised version of synX, the growth defect, which was detected in a glycerol-ethanol (YPGE) medium, was eliminated.

A second growth defect that was detected—this time in a glucose (YPD) medium at 30°C—was mapped to a reverse PCRtag within the designer sequence of the *FIP1* gene, causing 10 synonymously recoded codons (Wu *et al*. 2017). These erroneously recoded codons distorted the putative binding site for the Rap1 transcription factor, whose binding within transcription units is known to facilitate steric downregulation of gene expression. This, in turn, led to RNA polymerase stalling and a decrease in full-length transcript levels. When this error in the initial sequence design was corrected, the *FIP1*-related growth defect was reversed to the wild-type phenotype.

#### Synthetic chromosome synXII

*Saccharomyces cerevisiae*’s largest chromosome is chrXII, covering ∼20% of its 12.5 Mb genome. This chromosome is unique in that it includes ∼1.5 Mb of repetitive ribosomal gene clusters (rDNA), which encode ribosomal RNA (rRNA). A 976 067 bp synthetic version, synXII, was constructed by deleting 15 annotated repeat clusters, 28 introns and 21 tRNA genes; recoding of 123 TAG stop codons to TAA; and insertion of 299 loxPsym sites (Zhang *et al*. 2017). Several obstacles had to be overcome during the assembly of the synthetic DNA chunks in the initial semisynthetic synXII strains.

One essential tRNA gene, *TRR4* [*tR(CCG)L*], had to be restored with an ectopic copy thereof to bring the increased expression levels of genes involved in *S. cerevisiae*’s arginine metabolism (*ARG1*, *ARG4*, *ARG7* and *CPA2*) down to wild-type levels. It was hypothesised that the altered expression levels were related to the deficiency of leucyl tRNA. Second, transcript profiling indicated that the expression of three other genes—*CTR3*, *AHP1* and *GAS2*—was substantially altered in synXII strains. *CTR3* is located near a Ty insertion ‘hot spot’ in chrXII. It is therefore assumed that this gene is often mutated in many laboratory strains and that the deletion of the transposable elements near the 5΄ UTR of *CTR3* in synXII was responsible for the increased expression levels. It was also hypothesised that the downregulation of *AHP1* and *GAS2* observed in the synXII strains was a result of the incorporation of PCRtags or loxPsym sites.

The positioning of selective markers in each of the synthetic designer DNA megachunks was supposed to only interrupt non-essential genes; however, in many instances the markers weakened mitochondrial function or stress resistance. Another hurdle to overcome was that synonymous ORF-based recoding—introduced in synXII to accommodate one of the PCRtags, *YLL006W*—disrupted the function of the *MMM1* gene. And lastly, the omission of a presumed intron within the 5΄ UTR of *COQ9* resulted in a transcriptional blockage. Most of these defects were related to mitochondrial function. After a tedious process of identifying and debugging these glitches, the final updated version of the synXII-carrying strain displayed physiological fitness comparable to that of the wild-type parental strain.

Most interestingly, when a synthetically modified rDNA unit was used to replace the intact rDNA cluster and to regenerate rDNA at three distinct chromosomal locations, a well-organised nucleolus was still formed (Zhang *et al*. 2017). Furthermore, when *S. cerevisiae*’s 18S and 25S ‘internal transcribed spacer’ (ITS) regions of the rDNA unit (which is often used as a signature or barcode sequence in species identification) was replaced with the corresponding sequences of *S. bayanus*, cell growth was comparable to that of the wild-type strain. In other words, the rDNA locus of synXII can be moved to other chromosomal loci and a *cerevisiae/bayanus* chimeric ITS region within such a locus can support the formation of a normal nucleolar structure and wild-type cell growth in *S. cerevisiae*. It is important to note that chimeric ITS regions derived from either *Schizosaccharomyces pombe* or *Candida albicans* did not support normal nucleolar structure and wild-type cell growth in *S. cerevisiae* (Zhang *et al*. 2017). Nevertheless, the remarkable plasticity of the yeast genome along with the ability for intragenus ‘species morphing’ reflects a high degree of evolutionary flexibility by which these ITS ‘barcode’ regions can change within the same genus.

### Dotting 10 more i's before the Sc2.0 finish line

Finishing on the dot is our stated goal. With 30% of the work published, the Sc2.0 partners are leaving no stone unturned in their effort to complete the construction of the outstanding chromosomes by the end of this year. The full-length synthesis of the 10 remaining chromosomes is nearly finished. Most of the remainder of the laboratory work is about identifying the causes of fitness defects and the laborious process of the debugging of these flaws. This is a highly time-consuming and frustratingly labour-intensive component of the work. However, inspired by our progress, we refuse to see the difficulties in each of these 10 opportunities; rather we choose to see the opportunity in resolving the difficulties and challenges currently being encountered.

As the work on these 10 incomplete chromosomes progresses, the 6 full-length synthetic chromosomes are being used in parallel to test and interrogate other aspects of the Sc2.0 project. For example, as a first step towards constructing the ultimate Sc2.0 strain, functional double (synII plus synVI) and triple (synIII, synVI plus a linear version of synIXR) synthetic chromosome-carrying strains have been built (Mitchell *et al*. 2017). These synthetic chromosomes, which were constructed chunk by chunk in discrete strains, were consolidated in polysynthetic strains by ‘endoreduplication intercrossing’. The genome of the triple-synthetic strain is ∼6% synthetic overall, with ∼70 kb deleted, including 20 tRNA genes and >12 kb recoded. Genome sequencing of these polysynthetic strains indicates that suppressor mutations are not required to ensure co-existence of the Sc2.0 chromosomes. Only slightly slower growth rates were detected for the triple-synthetic strain, and these growth defects are likely to reflect deficits in tRNA abundance that will be corrected by introduction of the neochromosome, thereby clearing the way for completion of the ultimate designer synthetic yeast genome.

## CONNECTING DOTS FOR PIXEL-PERFECT BIG PICTURES OF SYNTHETIC CHROMOSOMES

Making sense of the dot matrix of data points and information generated by the Sc2.0 project and applying that knowledge judiciously to the design of industrially useful yeast cell factories will be like filling a grid of dots selectively to produce a pixel-perfect blueprint for every practical application.

Visualising a Hi-C three-dimensional (3D) conformation of the Sc2.0 chromosomes is a first step towards seeing the Big Picture of the Sc2.0 work (Mercy *et al*. 2017). As discussed above, the design of the synthetic Sc2.0 genome is conservative in terms of the preservation of the *S. cerevisiae*’s native gene content. However, the designer genome includes thousands of changes—changes to subtelomeric regions, recoding of TAG stop codons to TAA, relocation of the tRNA genes to a neochromosome, addition of recognisable PCRtag sequences, introduction of loxPsym sites, elimination of inconvenient restriction enzyme sites and deletions of repetitive sequences, including introns and transposons. An important piece of work was conducted to investigate whether these designer changes have altered the 3D organisation of synthetic and scrambled chromosomes (Mercy *et al*. 2017).

The global organisation of *S. cerevisiae*’s chromosomes is the result of the tethering and clustering of centromeres at the nuclear spindle pole body, as well as the non-rigid anchoring of small dynamic clusters of telomeres at the nuclear envelope (Duan *et al*. 2010; Mercy *et al*. 2017). The Hi-C conformation-capture approach enabled the comparison of the trajectories of synthetic chromosomes to those of their native counterparts in the yeast cell's nucleus, and these were found to be surprisingly similar. It is also known that the conformation of the nucleolus is influenced by the length of chromosomal arms, and remarkably, if the rDNA cluster is located much closer than usual to the spindle pole body, a massive reorganisation of many chromosomes occurs without a big impact on fitness (Mercy *et al*. 2017; Zhang *et al*. 2017). These 3D images of the synthetic chromosomes and the large data set obtained now and as future synthetic chromosomes are analysed, which will serve as a rich resource for future studies aimed at the effect of genome-wide engineering approaches of essential features of living systems.

## NEW BLUE AND OCHRE DOTS ON THE HORIZON

As the Sc2.0 project is making bold ideas in eukaryotic genome engineering go live, we are both inspired and challenged by the sheer number of dots emerging from our ‘blue-sky’ research and the pace at which they appear on our yeast canvass. We have come to learn that there is a whole spectrum of different tones and tints of dots—from blue to ochre—that are being discovered and creatively applied to enhance our painting of yeast cells. These dots vary from the finest of minute marks of new basic knowledge gained about yeast cells’ fundamentals neatly arranged on the canvas of the S288c laboratory strain of *S. cerevisiae* to bold, multicoloured dotting of innovative applications in industrial strains. The Sc2.0 work is, of course, creating only one of many dot-art images of yeasts in the global gallery of synthetic yeast genomics and semisynthetic yeast cell factories.

**Figure 7.**
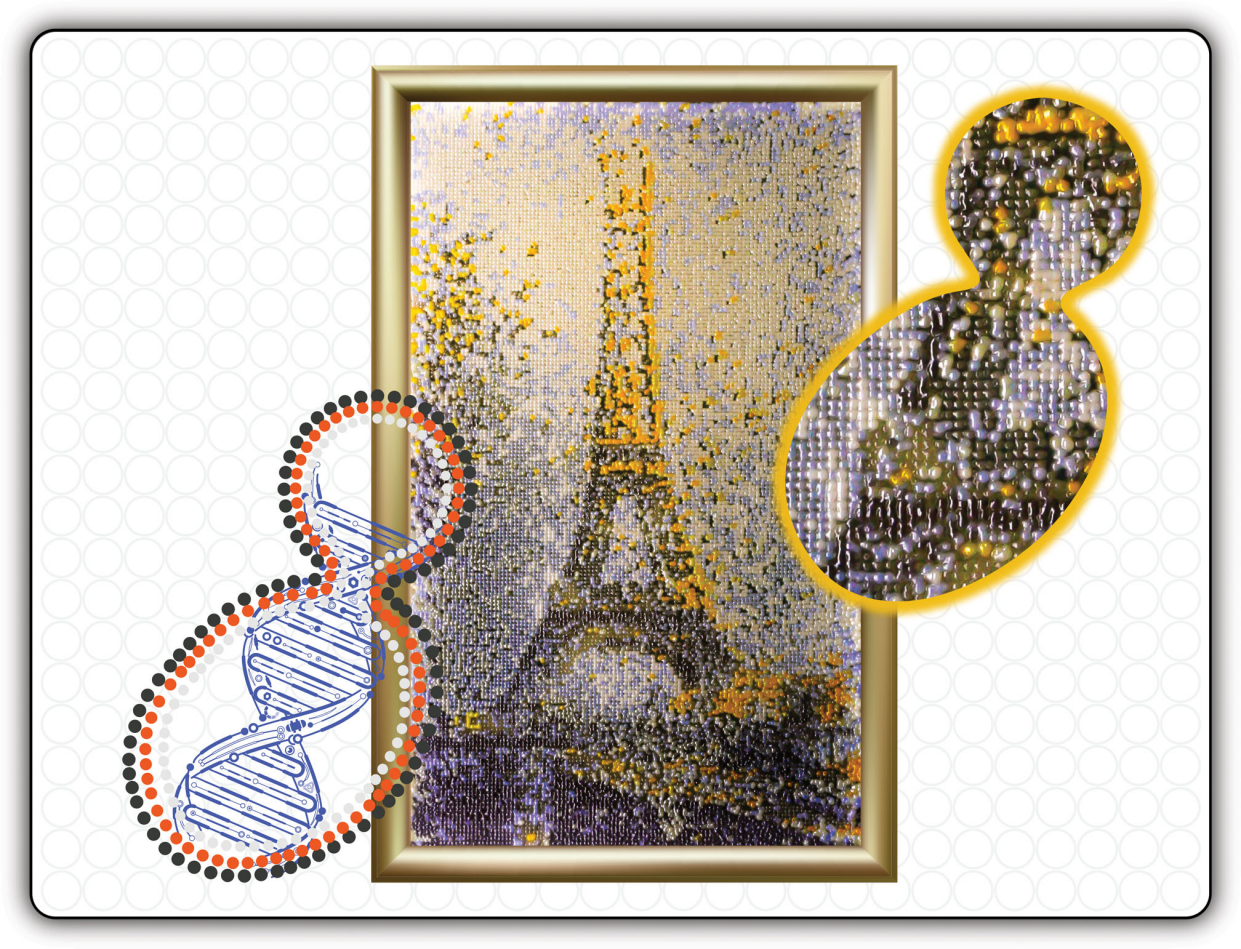
Yeast dot art is made by combining acoustic droplet ejection robotics with *Saccharomyces cerevisiae* engineered to produce diverse pigments. Design and layout by Jasmine Temple, NYU Langone Health (www.yeastart.org).

The Sc2.0 team also seeks to reach out to the public using the yeast as a kind of ‘dot-matrix printer’ to produce attractive and attention-grabbing artworks by literally combining thousands of dots, a form we sometimes call ‘biopointillism’. The individual dots consist of yeast engineered to produce colourful pigments of many hues (Fig. 7) and are arrayed using an acoustic droplet dispensing robot. This remarkable device can spray just under 25 000 2.5 nanolitre dots on a standard rectangular format agar plate. Within a few days, a colourful image appears! See www.yeastart.org for more examples of this entertaining application of synthetic yeast.

On a different note, one semisynthetic yeast cell factory that has attracted the bright spotlight of both the international community of synthetic yeast biologists and business leaders is the commercialised strain equipped to produce artemisinic acid, a precursor of the potent antimalarial compound artemisinin (Paddon *et al*. 2013). This work has inspired the construction of other industrially important yeasts, including a wine yeast capable of producing raspberry-flavoured Chardonnay (Lee *et al*. 2016) and improved yeast-based biosensors (Williams *et al*. 2016, 2017).

The work of the Sc2.0 consortium is laying history-making foundations upon which the world's grandest challenges can start to be addressed. How long will it be before the world could benefit from novel antibiotics, vaccines, biodegradable pesticides and energy-rich chemicals from semisynthetic yeasts containing minimalist genomes? How far are we from constructing a functional *S. cerevisiae* genome consisting of a single synthetic chromosome?

Connecting the dots and mapping a future can be tricky. In this article, we offered some optimistic future predictions with humility balanced by the reality that the uncertainties beyond the immediate horizon of a successful Sc2.0 project outcome can become overwhelming. Who knows what the future of synthetic genomics might look like in few years’ time? All that we can say at this stage is ‘watch this space *dot, dot, dot*’.
